# CDC25B induces cellular senescence and correlates with tumor suppression in a p53-dependent manner

**DOI:** 10.1016/j.jbc.2021.100564

**Published:** 2021-03-18

**Authors:** Ying-Chieh Chen, Hsi-Hsien Hsieh, Hsi-Chi Chang, Hsin-Chiao Wang, Wey-Jinq Lin, Jing-Jer Lin

**Affiliations:** 1 Institute of Biopharmaceutical Sciences, National Yang-Ming University, Taipei, Taiwan; 2 Institute of Biochemistry and Molecular Biology, National Taiwan University College of Medicine, Taipei, Taiwan

**Keywords:** senescence, CDC25B, p53, tumor suppression, phosphatase, Cdc25B, cell division cycle 25B, DDR, DNA damage response, FBS, fetal bovine serum, OIS, oncogene-induced senescence, PBS, phosphate buffered saline, PCPG, pheochromocytoma and paraganglioma, pNPP, *p*-nitrophenyl phosphate, RIPA, radioimmunoprecipitation assay, TCGA, The Cancer Genome Atlas

## Abstract

The phosphatase cell division cycle 25B (Cdc25B) regulates cell cycle progression. Increased Cdc25B levels are often detected in cancer cell lines and human cancers and have been implicated in contributing to tumor growth, potentially by providing cancer cells with the ability to bypass checkpoint controls. However, the specific mechanism by which increased Cdc25B impacts tumor progression is not clear. Here we analyzed The Cancer Genome Atlas (TCGA) database and found that patients with high CDC25B expression had the expected poor survival. However, we also found that high CDC25B expression had a p53-dependent tumor suppressive effect in lung cancer and possibly several other cancer types. Looking in more detail at the tumor suppressive function of Cdc25B, we found that increased Cdc25B expression caused inhibition of cell growth in human normal fibroblasts. This effect was not due to alteration of specific cell cycle stage or inhibition of apoptosis, nor by induction of the DNA damage response. Instead, increased CDC25B expression led cells into senescence. We also found that p53 was required to induce senescence, which might explain the p53-dependent tumor suppressive function of Cdc25B. Mechanistically, we found that the Cdc25B phosphatase activity was required to induce senescence. Further analysis also found that Cdc25B stabilized p53 through binding and dephosphorylating p53. Together, this study identified a tumor-suppressive function of Cdc25B that is mediated through a p53-dependent senescence pathway.

Cell division cycle 25B (Cdc25B) phosphatase is a key player in G2/M cell cycle progression (1). The Cdc25B protein level begins to increase from mid S-phase and peaks during the G2/M phase (2). It then activates the Cdk1-cyclinB1 complexes during the G2 to M phase transition of the cell cycle (3, 4). The Cdc25B protein level is rapidly decreased by the proteasome pathway after M phase of the cell cycle (2, 5). Cdc25B protein level was also found to be increased after DNA damages such as UV radiation, ionizing radiation, or genotoxic agents. The increased Cdc25B level has a role in recovery from the G2/M checkpoint activated in response to DNA damages (6, 7). It leads to bypass of genotoxic-induced G2/M checkpoint arrest.

In addition to cell cycle deregulation, CDC25B overexpression also has an oncogenic property. It was shown that Cdc25B cooperated with either oncogenic HRAS or Rb inactivation to transform mouse embryonic fibroblasts *in vitro* and to form tumors *in vivo* (8). It was also reported that targeted overexpression of CDC25B in mammary glands of mice induced hyperplasia and promoted proliferation (9). Tumor growth could then be induced with additional challenge by carcinogen (10). Thus, although CDC25B overexpression is not sufficient to induce tumor growth, it impacts positively toward tumorigenesis.

CDC25B has been reported to be overexpressed in various cancer cell lines and human cancers (11, 12). Although the mechanism of how CDC25B expression is increased in cancer cells is still unclear, several lines of evidence showed that it might be related to oncogene activation and/or p53 inactivation. For example, it was shown that CDC25B mRNA level was increased in SV40- or HPV E6 and E7-transformed fibroblasts (11). CDC25B was also shown to be a target of both oncogene c-myc and tumor suppressor gene p53 (13, 14, 15). With its role in checkpoint control, Cdc25B overexpression might contribute to tumorigenic pathway by rapidly pushing cells into mitosis with incompletely replicated DNA, thus providing cancer cells with the ability to bypass checkpoint control to enhance their proliferation.

To reveal the impact of increased Cdc25B in tumorigenesis, we first analyzed the association of CDC25B expression to survival of cancer patients. To our surprise, we found that high CDC25B expression significantly associated with better survival for patients carried wild-type p53 in lung cancer (LUNG) and possibly several other cancer patients. The results suggested that excess Cdc25B has a p53-dependent tumor-suppressive role. To evaluate the cellular effects of increased Cdc25B, we applied normal human fibroblasts in our analysis. We found CDC25B overexpression induced senescence in a p53-dependent manner. The observed phenomenon did not appear to be limited to normal lung fibroblast as skin fibroblast and several cancer cells also showed similar effects. We further showed that Cdc25B stabilized p53 protein through interacting with p53 and dephosphorylated p53. Thus, our results provide evidence to support a role of Cdc25B in senescence induction that is related to tumor suppressing.

## Results

### High CDC25B expression is beneficial to cancer patients with wild-type p53

**Figure 1:**
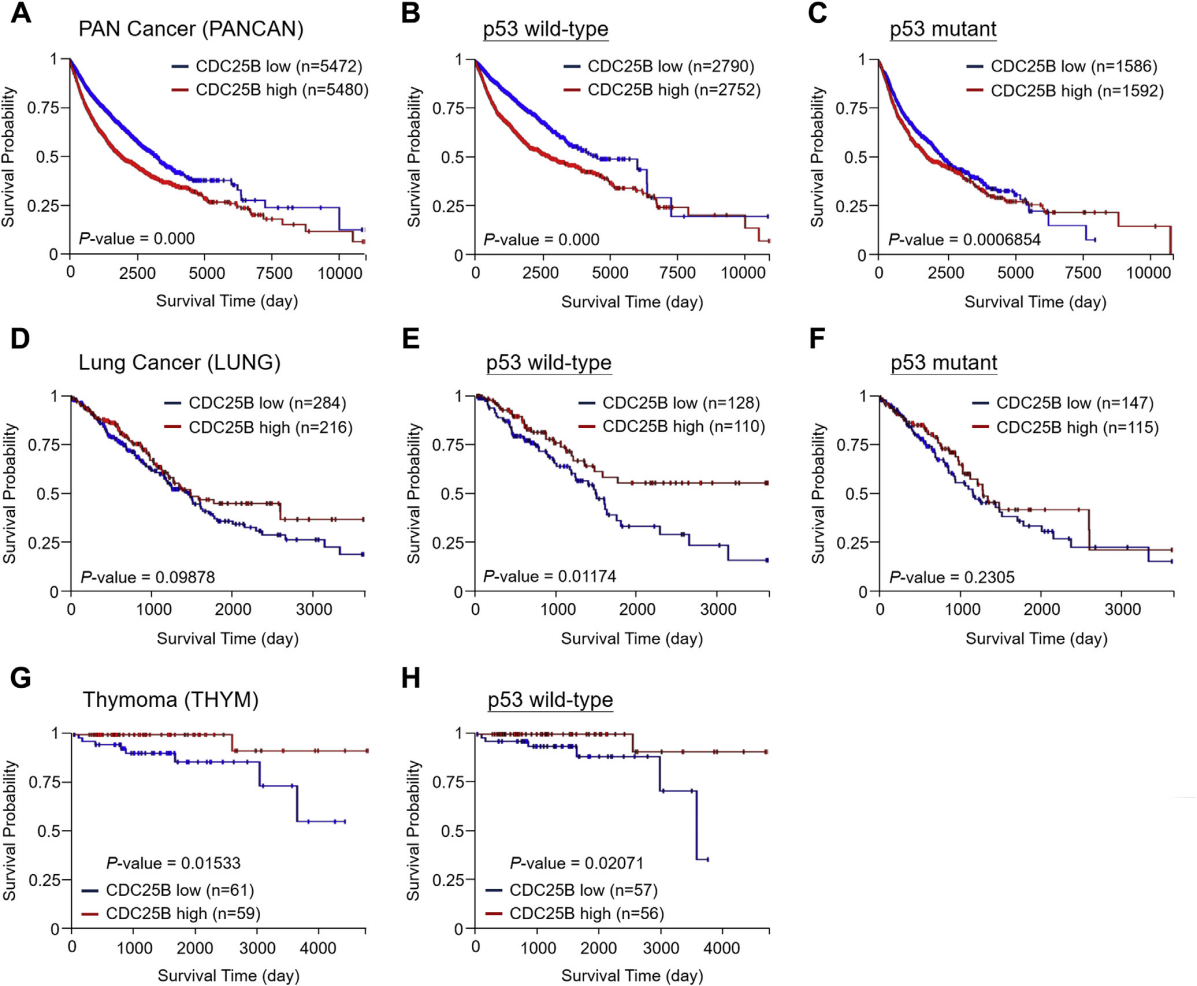
**Kaplan–Meier survival curves of CDC25B expression levels and TP53 mutation status.** The UCSC Xena (http://xena.ucsc.edu/) software was used to analyze data set provided by TCGA of National Cancer Institute (USA) (https://www.cancer.gov/about-nci/organization/ccg/research/structural-genomics/tcga). *A*, data from the TCGA Pan-Cancer (PANCAN) study were analyzed. In the analysis, CDC25B expression levels were broadly divided into two groups high (*red*) and low (*blue*). *B*, the wild-type p53 cancers in the PANCAN study were analyzed. *C*, mutant p53 cancers in the PANCAN study were analyzed. *D*, data from the TCGA Lung cancer (LUNG) study were analyzed. CDC25B expression levels were broadly divided into two groups high (*red*) and low (*blue*). *E*, the wild-type p53 cancers in the LUNG study were analyzed. *F*, mutant p53 cancers in the LUNG study were analyzed. *G*, the TCGA Thymoma (THYM) study was analyzed as above. *H*, the wild-type p53 cancers in the THYM study were analyzed. The statistic values (*P*) and number of patients (n) are indicated.

Overexpressed CDC25B has been reported in various cancer cell lines and human cancers (11, 12). To determine the role of increased Cdc25B in tumorigenesis, the association of CDC25B expression determined by RNA-seq analysis to the survival of cancer patients was analyzed. Data set provided by the TCGA program of National Cancer Institute (USA) (https://www.cancer.gov/about-nci/organization/ccg/research/structural-genomics/tcga) was applied in the analysis. The Kaplan–Meier plot analyses were conducted using UCSC Xena software (http://xena.ucsc.edu/). The survival for the TCGA Pan-Cancer data set (PANCAN) was first analyzed. Patients were separated into high and low CDC25B-expression groups. The analyses showed that high CDC25B expression associated with poor survivals (Fig. 1*A*). With the close association between p53 and CDC25B expression, the p53 mutation status was also added to the analysis. We found high CDC25B expression associated with poor survivals regardless of the p53 status (Fig. 1, *B* and *C*). The results suggest that high CDC25B expression impacts negatively to the overall survival of cancer patients, consistent with the expected oncogenic role of CDC25B.

Next, the impact of CDC25B expression and p53 mutation status on individual cancer types was analyzed. Interestingly, we found that CDC25B expression did not appear to have an impact on the overall survival of lung cancer (LUNG) patients (Fig. 1*D*). Surprisingly, when lung cancer patients were grouped based on their p53 status, high CDC25B expression significantly associated with better survival in patients carried wild-type p53 (Fig. 1, *E* and *F*). High CDC25B expression level also showed better survival probability in thymoma (THYM) patients carried wild-type p53 (Fig. 1, *G* and *H*). Moreover, similar association could be observed in several other cancer types, although the statistical analysis did not show significance due to the limited number of patients. For example, a similar trend could be observed for colon and rectal cancer (COADREAD) patients (Fig. S1, *A*–*C*). High CDC25B expression levels might also beneficial to the overall survivals of pheochromocytoma and paraganglioma (PCPG) and testicular cancer (TGCT) patients (Fig. S1, *D* and *E*). Thus, in addition to the oncogenic function, the analysis identified a p53-dependent tumor-suppressive role of Cdc25B in several cancer types.

### Overexpression of Cdc25B induces senescence in normal human fibroblasts

**Figure 2:**
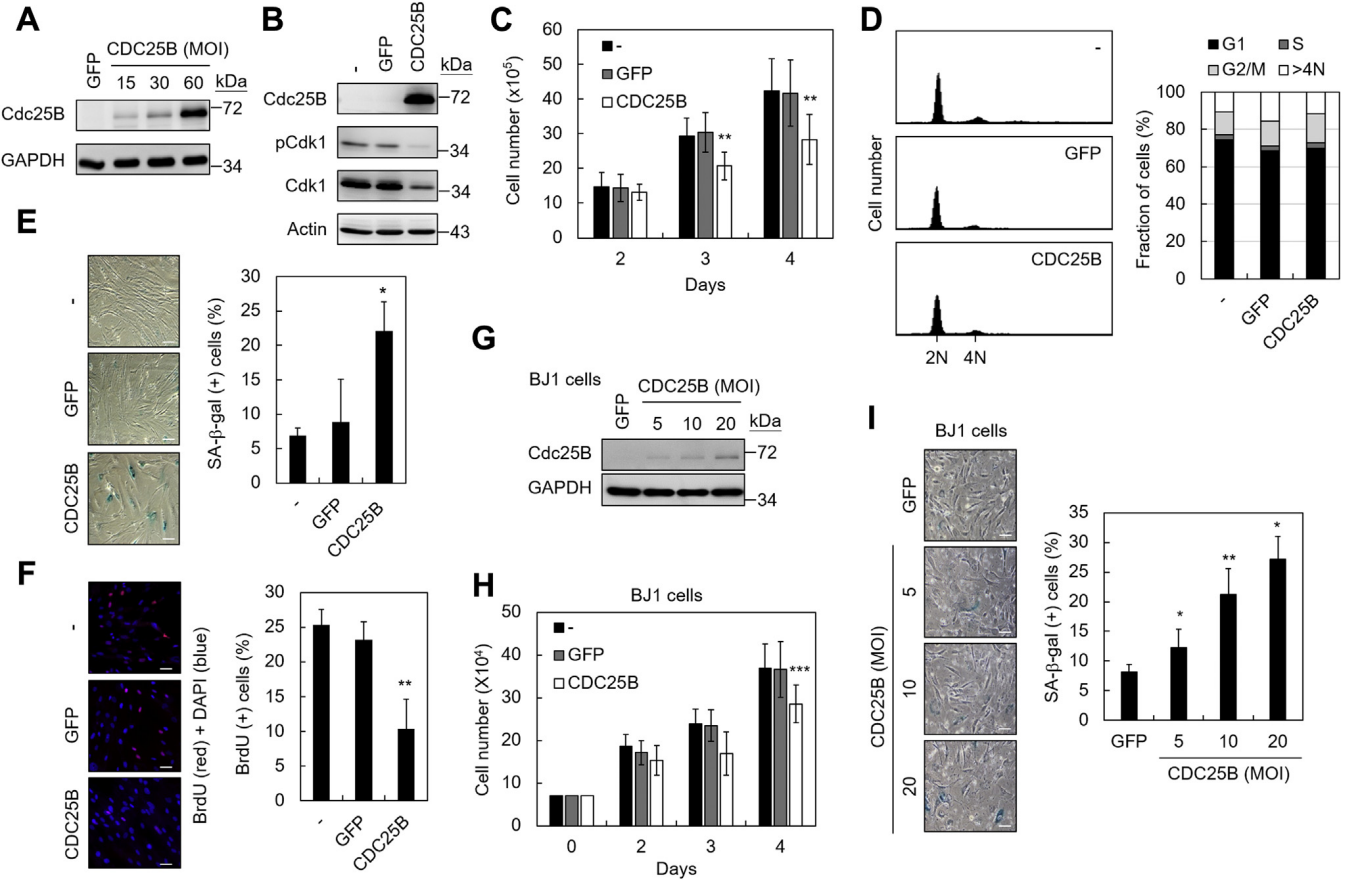
**CDC25B overexpression induced senescence in normal human fibroblasts.***A*, IMR90 cells were infected with adenoviruses carrying CDC25B or GFP. Cell extracts were prepared 3 days after infection (MOI = 15, 30, or 60) and then analyzed by immunoblotting assays using antibodies against Cdc25B or GAPDH. *B*, IMR90 cells were infected without (−) or with adenoviruses carrying CDC25B or GFP at MOI = 60 and then analyzed for Cdk1 and phosphorylated Cdk1 (Tyr15) by immunoblotting assays. *C*, as above, the cell numbers were determined 2, 3, or 4 days after infection using trypan blue assay. *D*, the cell cycle distribution of Cdc25B-expressing IMR90 cells was determined 3 days after infection. The flow cytometry histograms of the PI-stained cells are presented (*left panel*). Quantification of different cell cycle phases is also presented (*right panel*). *E*, three days after infection, the cells were analyzed for SA-β-gal. Photographs of the X-gal stained cells are shown. *Right panel* shows the quantification of the SA-β-gal-positive cells. *F*, three days after infection, the CDC25B- or GFP- expressing IMR90 cells were cultured in medium containing 40 μM BrdU for 16 h and then fixed and stained with anti-BrdU antibody (*red*). The nuclei were stained by DAPI (*blue*). *Right panel* shows the quantification of the BrdU- and DAPI-positive cells. *G*, normal skin fibroblast BJ1 cells were infected with adenovirus carrying GFP (MOI = 20) or CDC25B (MOI = 5, 10, or 20). Total cell extracts were prepared 3 days after infection and analyzed by immunoblotting assays using antibodies against Cdc25B or GAPDH. *H*, BJ-1 cells were infected with adenovirus carrying GFP or CDC25B at MOI = 20. The cell numbers were determined using trypan blue assays. *I*, as above, the senescence phenotype was analyzed by SA-β-gal assays 3 days after infection. The percentages of SA-β-gal-positive cells were quantified. Quantification of results present in (*D*, *E*, *F*, *H* and *I*) were conducted from 3 to 4 independent experiments. Student’s *t*-test was applied for statistical analysis. Values with *p* < 0.05, 0.01, or 0.001 were marked with ∗, ∗∗, or ∗∗∗, respectively. In (*E*, *F* and *I*), the scale bar represents 50 μm.

The mechanism of how increased Cdc25B rendered its tumor-suppressive effect was analyzed. Previous analyses had shown that increased Cdc25B caused diverse and sometimes opposite effects in different cancer cells (16, 17). To avoid the genetic diversity of cancer cells, human normal lung fibroblast (IMR90) cell was selected in this study. The Cdc25B level in IMR90 cells was low or undetectable using immunoblotting assays (Fig. 2*A*). Due to the low delivery efficiency of gene into normal fibroblasts, adenoviral expression system was applied to introduce CDC25B into IMR90 cells. Still, high viral titers were required to express detectable Cdc25B (Fig. 2*A*). The multiplicity of infection (MOI) value equivalent to 60 was chosen for subsequent experiments as this MOI efficiently expressed CDC25B or GFP. The expressed CDC25B was functional as it reduced the Cdk1 phosphorylation at Tyr15 and reduced Cdk1 level (Fig. 2*B*). Thus, CDC25B was successfully introduced into normal human fibroblast IMR90 cells using the adenoviral delivery system.

Proliferation of IMR90 cells was first evaluated by counting the cell numbers after CDC25B overexpression. As shown in Figure 2*C*, the growth rate of CDC25B-overexpressing cells was decreased, indicating that high Cdc25B level inhibited the proliferation of normal cells. With the role of Cdc25B in cell cycle regulation, the effect of increased Cdc25B on cell cycle progression was analyzed. Flow cytometry analysis showed that excess amount of Cdc25B did not appear to cause a significant change in the distribution of cell cycle phases (Fig. 2*D*). It was also apparent that the number of sub-G1 cells was not increased upon CDC25B overexpression, suggesting that increased Cdc25B does not cause apoptosis in normal fibroblasts.

The morphology of the CDC25B-overexpressing cells appeared to be enlarged and flattened, a phenotype similar to that of senescent cells (Fig. 2*E*). Thus, the senescence-associated features in CDC25B-overexpressing cells were then assessed (18, 19). The CDC25B-overexpressing cells showed an increase of the SA-β-gal activity (Fig. 2*E*). The percentage of SA-β-gal-positive cells was increased from <10% in normal or GFP-expressing cells to ∼25% in CDC25B-overexpressing cells. Consistent with this observation, the proportion of BrdU-positive cells was reduced from ∼25% in normal or GFP-expressing cells to ∼10% in CDC25B-overexpressing cells (Fig. 2*F*). The growth-inhibitory and senescent phenotypes were not limited to normal lung fibroblast, as human normal skin fibroblast BJ1 also showed growth inhibition and activation of the SA-β-gal activity upon CDC25B overexpression (Fig. 2, G–I). Together, the results show that a senescent program is initiated in response to CDC25B overexpression in normal fibroblasts.

It was reported that senescent cells often upregulate enzymes that degrade the extracellular matrix and secrete immune modulators and inflammatory cytokines to reinforce senescence in an autocrine and paracrine manner (20). The senescence-associated secretory phenotype (SASP) might heighten inflammation through recruitment of inflammatory cells and thus has detrimental effects on the tissue microenvironment through alteration of tissue composition and architecture. Interestingly, the signature SASP components, including IL-1α, IL-1β, IL-6, IL-8, Wnt2, TGF-β1, TGF-β3, serpinB2, and serpinE2, did not appear to be induced in CDC25B-overexpressing cells (Fig. S2). The results suggest that although a senescent program is induced, it is distinctly different from replicative senescence.

### The Cdc25B activity is required to induce senescence in human normal fibroblasts

**Figure 3:**
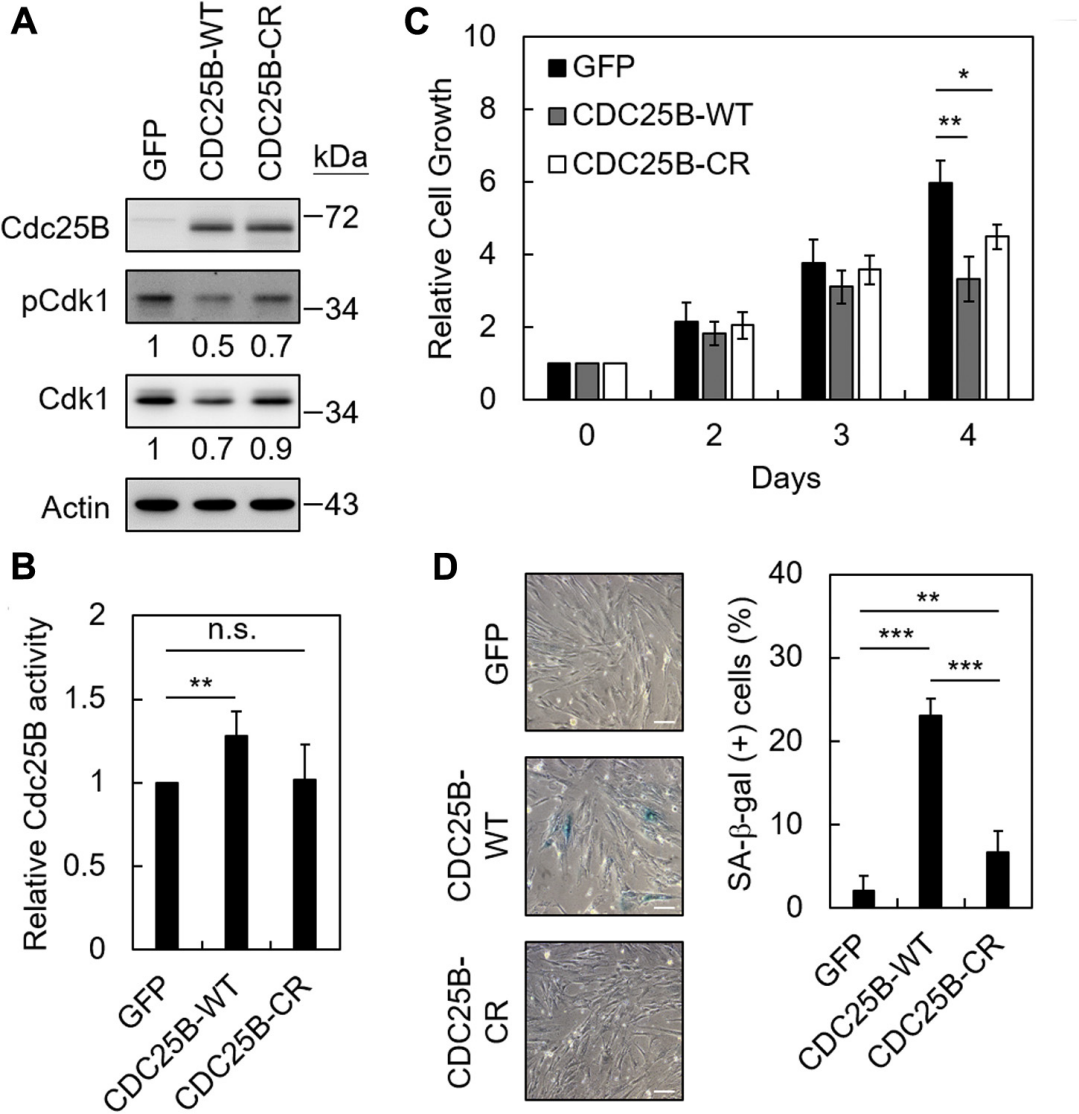
**The Cdc25B phosphatase activity is required for inducting senescence.***A*, IMR90 cells were infected with adenoviruses carrying GFP, CDC25B-WT, or CDC25B-CR mutant at MOI = 60. Cell extracts were prepared 3 days after infection and then analyzed by immunoblotting assays. *B*, as above, cell extracts were prepared from IMR90 cells expressing GFP, CDC25B-WT, or CDC25B-CR. Cell extracts were then immunoprecipitated by anti-Cdc25B antibodies and analyzed by phosphatase activities using pNPP as substrate. Relative Cdc25B activities from six independent experiments are presented using the activity of cells expressing GFP as 1. *C*, the cell numbers of GFP-, CDC25B-WT-, or CDC25B-CR-expressing cells were determined by counting the cell numbers using trypan blue-staining at days 2, 3, and 4 post transfection. The relative growths from the average of four independent experiments are presented. *D*, three days after infection, the cells were analyzed for SA-β-gal activity. Photographs of the X-gal stained cells and quantification of the SA-β-gal positive cells from five independent experiments are presented. The scale bar represents 50 μm. Student’s *t*-test was applied for statistical analysis. Values with *p* < 0.05, 0.01, or 0.001 were marked with ∗, ∗∗, or ∗∗∗, respectively.

Cdc25B is a protein phosphatase, to test whether the enzymatic activity is important for senescence, a CDC25B-CR mutant carrying both C487S and R493K (CR) mutations was constructed. It was reported that Cdc25B carrying mutations of these two residues lost its phosphatase activity (21). Adenovirus carrying this mutant was generated and introduced into IMR90 cells. As shown in Figure 3*A*, while the wild-type CDC25B reduced both the phosphorylation and protein level of Cdk1, the CDC25B-CR mutant did not. The Cdc25B phosphatase activity within IMR90 cells was also analyzed. Total cell extracts were prepared from CDC25B-WT- or CDC25B-CR-expressing cells, immunoprecipitated with anti-Cdc25B antibody, and then analyzed for the phosphatase activities. As shown in Figure 3*B*, although the phosphatase activity was increased in CDC25B-WT-expressing cells, no elevation of phosphatase activity was observed in CDC25B-CR-expressing cells. These results indicated that the Cdc25B-CR mutant indeed lost its phosphatase activity. The proliferation and SA-β-gal activity were then analyzed in CDC25B-CR-expressing cells. As shown in Figure 3, *C* and *D*, cells expressing the Cdc25B-CR mutant showed moderate growth inhibition and did not show apparent SA-β-gal-inducing activities. The results suggest that the phosphatase activity is important for Cdc25B-mediated senescence.

### p53 is required for Cdc25B-induced senescence

**Figure 4:**
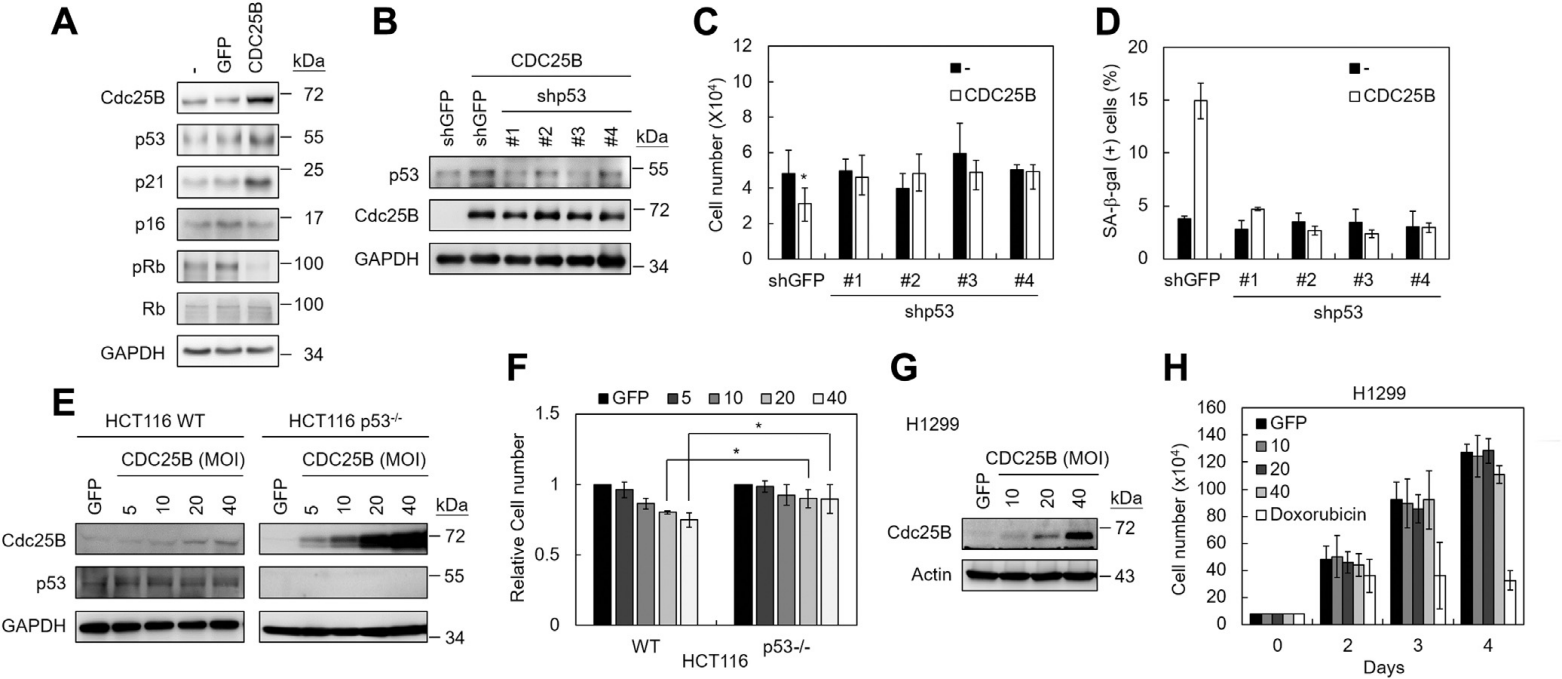
**p53 is required for Cdc25B-induced senescence in normal human fibroblasts.***A*, IMR90 cells were infected with adenoviruses carrying GFP or CDC25B. Cell extracts were prepared 2 days after infection and then analyzed by immunoblotting assays. *B*, IMR90 cells were infected with lentiviruses carrying shRNA against p53 or GFP. The p53-knocked down IMR90 cells were then infected by adenovirus carrying CDC25B. Three days after infection, cell extracts were prepared and then analyzed by immunoblotting assays using antibodies against p53, Cdc25B, and GAPDH. *C*, as above, the cell numbers were measured 3 days after infection. *D*, the SA-β-gal positive cells were determined in CDC25B overexpressing cells that were knocked down p53. Quantification of SA-β-gal positive cells was presented 3 days after infection. *E*, HCT116 WT or p53^−/−^ cells were transfected with indicated MOI of adenovirus carrying CDC25B. The cell extracts were prepared 3 days after infection and analyzed by immunoblotting assays. *F*, the cell numbers were also determined. *G*, H1299 cells were transfected with indicated MOI of adenovirus carrying CDC25B. The cell extracts were prepared 3 days after infection and analyzed by immunoblotting assays. *H*, the cell numbers were also determined at days 2, 3, and 4 post transfection. Doxorubicin was used as a control. In (*C*, *D*, *F* and *H*), Student’s *t*-test was applied for statistical analysis from three independent experiments. Values with *p* < 0.05 were marked with ∗.

To gain insight into the molecular basis of Cdc25B-induced senescence, the expressions of several senescent mediators were examined using immunoblotting assays. In IMR90 cells, CDC25B overexpression increased both p53 and p21 levels and decreased Rb protein phosphorylation (Fig. 4*A*). To investigate the involvement of p53 in Cdc25B-induced senescence, we applied RNA interference approach to knock down p53 level using lentiviral delivery system (22). The IMR90 cells were first transduced with lentivirus expressing short hairpin RNAs (shRNAs) targeting p53 or GFP and then infected with adenovirus-carrying CDC25B. As shown in Figure 4*B*, the level of p53 was reduced upon shRNA treatments. The growth and SA-β-gal activity of these cells were also analyzed. In p53 knockdown cells, the growth-inhibitory activity of Cdc25B was disappeared and the number of SA-β-gal-positive cells was also decreased (Fig. 4, *C* and *D*). The results indicate that p53 is required for Cdc25B-induced senescence.

The p53-dependent cellular effect of Cdc25B was also evaluated in cancer cells. Two colorectal cancer cell lines, HCT116 and its isogenic p53-deleted cell, were adopted in our studies (23). CDC25B or GFP was introduced into these cells and examined their growth (Fig. 4*E*). We found that the growth of p53 wild-type cells was inhibited upon CDC25B overexpression, but not in the p53^−/−^ deleted cells (Fig. 4*F*). We also found that overexpressing CDC25B did not inhibit cell growth in lung cancer H1299 cell that lacks p53 (Fig. 4, *G* and *H*). Thus, the observed p53-dependent growth inhibitory effect of Cdc25B is not limited to normal fibroblasts.

### Excess Cdc25B binds and stabilizes p53 protein

CDC25B overexpression was reported to cause centrosome overduplication through stabilizing centrin 2 (24, 25). Abnormal centrosome duplication then induces DNA damage response (DDR) (26). Since p53 is a key mediator of DDR, Cdc25B may upregulate p53 through inducing DDR. To test this possibility, the DDR was evaluated in CDC25B expressing cells. Phosphorylation of H2AX (γ-H2AX) and several transducers (ATM, ATR, CHK1, and CHK2) were used as criteria for DDR activation. As shown in Fig. S3, CDC25B overexpression did not cause accumulation of γ-H2AX DNA damage foci, nor activated DDR transducers, suggesting that the increased p53 level was not due to DDR induction.

**Figure 5:**
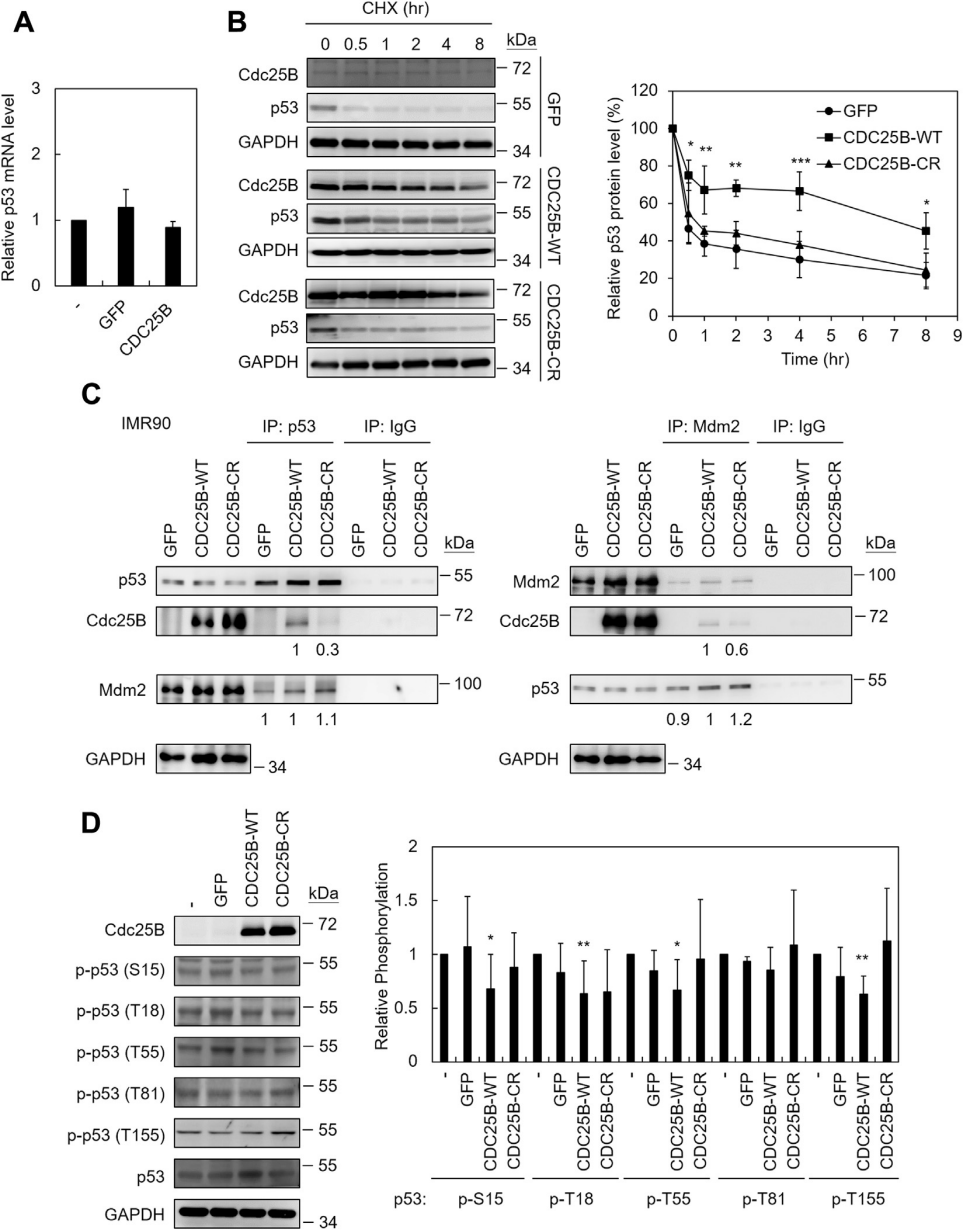
**Cdc25B binds to p53 and stabilizes p53.***A*, the mRNA from GFP- or CDC25B-infected cells was prepared 3 days after infection and then analyzed by reverse transcription (RT)-q-PCR. The averages of five independent experiments are presented. *B*, IMR90 cells expressing GFP, CDC25B-WT, or CDC25B-CR were incubated with 20 μg/ml cycloheximide (CHX) and then analyzed by immunoblotting assays at indicated time (*left panel*). Quantification of p53 levels from six independent experiments was determined (*right panel*). Student’s *t*-test was applied to compare the values of GFP and CDC25B-WT groups. Values with *p* < 0.05, 0.01, or 0.001 were marked with ∗, ∗∗, or ∗∗∗, respectively. *C*, three days after infection, cell extracts prepared from Cdc25B-expressing cells were immunoprecipitated with IgG, anti-p53 (*left panel*), or anti-Mdm2 (*right panel*) antibodies. The precipitated proteins were analyzed by immunoblotting assays. Relative Cdc25B, Mdm2, or p53 values in the immunoprecipitates were presented. The numbers were obtained from three independent experiments, normalized with the immunoprecipitates of p53 (*left panel*) or Mdm2 (*right panel*), and then used CDC25B-WT as 1. *D*, as above, cell extracts prepared from IMR90 cells expressing GFP, CDC25B-WT, or CDC25B-CR were analyzed by immunoblots (*left panel*). Quantification of p53 phosphorylation at indicated residues from 3 to 5 independent experiments is presented. Values with *p* < 0.05 or 0.01 were marked with ∗ or ∗∗, respectively.

Accumulation of p53 could be caused by increasing p53 expression and/or reducing p53 degradation. The expression of p53 at the transcription level was first measured. We found that CDC25B-overexpression did not affect the mRNA level of p53 (Fig. 5*A*). The effect of Cdc25B on the stability of p53 protein was next analyzed. The p53 is a short-lived protein with a half-life <30 min (Fig. 5*B*). We found that Cdc25B stabilized p53 by extending its half-life to ∼6.5 h. The stability of p53 was also determined in cells carrying the CDC25B-CR mutant. We found that the Cdc25B-CR mutant did not alter the half-life of p53 protein (Fig. 5*B*), indicating that the phosphatase activity of Cdc25B is required for p53 stabilization. The protein stability of both wild-type and the Cdc25B-CR mutant was also determined. We found that the stability of these two proteins was similar (Fig. S4), suggesting that failed stabilization of p53 was not due to reduced stability of the Cdc25B-CR mutant protein.

Control of p53 stability is mainly regulated by the proteasomal degradation pathway (27). Mdm2 (mouse double minute 2) is the primary E3 ligase responsible for p53 degradation. The possibility of Cdc25B in interfering with p53 turnover pathway was tested. We first analyzed the interaction of Cdc25B to p53 and Mdm2. Co-immunoprecipitation experiments showed that Cdc25B was detected in the immuneprecipitates of both p53 and Mdm2, indicating that Cdc25B interacted with both p53 and Mdm2 (Fig. 5*C*). Quantification of the immunoprecipitates also showed that Cdc25B did not appear to uncouple the interaction between p53 and Mdm2. Moreover, the Cdc25B-CR mutant levels were reduced in the immunoprecipitates of both p53 and Mdm2, suggesting that the binding of Cdc25B-CR to p53 and Mdm2 was reduced. The result is consistent with an earlier report that the Arg493 residue of Cdc25B is important for substrate recognition (28) and implicated that Cdc25B might target p53 directly.

Phosphorylation of p53 has a critical role in regulating protein stability (27). To test whether Cdc25B regulates p53 stability through dephosphorylating p53, the phosphorylation status of p53 on several Ser and Thr residues was analyzed. Specifically, Thr18 (29), Thr55 (30, 31, 32), Thr81 (33), and Thr155 (34) were selected for this analysis for their known roles in regulating p53 stability and the availability of specific antibodies. Because Ser15 was reported to be important for Thr18 phosphorylation, the status of Ser15 phosphorylation was also analyzed (29, 35). As shown in Figure 5*D*, moderate reduction of p53 phosphorylation was observed on residues S15, T18, T55, and T155 in cells overexpressing CDC25B. In contrast, the CDC25B-CR mutant did not have an impact on p53 phosphorylation. The results indicated that the phosphatase activity of Cdc25B is required for reducing p53 phosphorylation at these residues. Since more than one Ser/Thr residues showed reduced phosphorylation, the results also suggested that Cdc25B did not act on a specific residue. Together, these results provide evidence for the function of Cdc25B in senescence that it binds and dephosphorylates p53, thus stabilizing p53 protein. Accumulation of p53 then activates the senescence pathway.

## Discussion

Excess Cdc25B has been implicated in having a function as an oncogene that impacts positively to cancer cell progression during tumorigenesis (8, 12). TCGA database analysis supports the expected role of high CDC25B in tumorigenesis. However, the analysis also revealed that high Cdc25B levels have better survivals in lung and several other cancers, suggesting a tumor-suppressive function of Cdc25B. The finding of senescence induction by increased CDC25B expression provides an explanation to the tumor-suppressive role of Cdc25B. However, this finding did not fully explain the mechanism of how the tumor-suppressive effect was only observed in a subset of cancer types. It has been well recognized that the number of mutations and the mutated genes varies across tumor types. These cancer-type-specific mutations have their specific effect on survivals (36). Thus, although the reason is still unclear to us, it is likely that more factor is required for this cancer-type-specific tumor suppressive function of Cdc25B.

Senescence is generally recognized as a barrier against tumorigenesis (37). Escape of senescence is thought to be required for tumor progression. Since Cdc25B level could be induced by transforming normal fibroblasts with c-myc, SV40, or HPV E6 and E7 (11, 13), the increased Cdc25B might trigger the senescence pathway to prevent transformation of normal cells in early stage of tumorigenesis. Thus, elevation of Cdc25B in early tumor development is likely to be tumor suppressive. Overcoming senescence during early tumor progression may be difficult, as it requires the inactivation of p53. However, chromosome abnormalities accumulated in CDC25B-overexpressing cells might increase the possibility of p53 inactivation to promote further tumor progression. Since p53 was reported to be a negative regulator of CDC25B (14, 15), inactivation of p53 might further increase the expression of CDC25B for later tumor development. The increased Cdc25B is then oncogenic that it enhances cancer cell proliferation through bypassing checkpoint control. The scenario might explain the dual function of Cdc25B during tumor development.

With the role of Cdc25B in cell cycle regulation, increased CDC25B should, in theory, cause replicative stress and genomic instability. It is thus anticipated that cellular stress response should be induced in order to respond to aberrant cell cycle progression. However, the cellular response upon CDC25B overexpression appears to be very diverse in earlier studies. For example, overexpression of CDC25B was shown to increase apoptosis after radiation in TE8 esophageal cancer cells (16); yet in skin squamous cell carcinoma cell, overexpression of CDC25B appeared to inhibit apoptosis (17). Since these studies were conducted in cancer cells that have accumulated large chromosome aberrations, the cellular effects of CDC25B overexpression might depend on specific cellular context of cancer cells. Thus, the role of excess Cdc25B on tumorigenesis cannot not be fully determined using cancer cell lines. In this study, we found that Cdc25B induced senescence in the context of normal cells. The observation is distinctly different from the apoptotic-related cellular effects in cancer cells. Thus, application of normal fibroblasts could provide additional assessment for the contribution of CDC25B during tumor development.

Overexpression of oncogenes in normal fibroblasts was shown to induce senescence (38). This phenomenon is termed oncogene-induced senescence (OIS). A notable example is the induction of senescence by overexpressing oncogenic *ras* in both human and rodent normal fibroblasts (39). Although OIS could be induced by overexpressing various types of oncogenes, p53 appears to be a common mechanism to mediate activation of the DDR pathway (40). Taking *ras* as an example, it was reported that *ras* overexpression triggered reactive oxygen species production and/or DNA hyperreplication to stimulate a p53-dependent DDR (41, 42). Thus, the finding of p53 dependence in CDC25B-induced senescence implicates that a similar DDR pathway might be activated. However, we found increased Cdc25B did not perturb cell cycle, nor did it activate DDR. Instead, we found that Cdc25B binds to p53 to dephosphorylate and stabilize p53. These results provide evidence that the Cdc25B might act directly on p53 to trigger senescence pathway. We also noted that unlike OIS, the SASP was not identified in cells with increased Cdc25B. Thus, senescence induced by CDC25B overexpression is mechanistically different from conventional OIS.

The stability of p53 is mainly regulated by ubiquitin-dependent degradation pathway (43). Through the binding to specific ubiquitin-ligase Mdm2, p53 is ubiquitinated and subsequently degraded by the proteasome. Phosphorylation also plays important role in regulating the stability of p53 (27). For example, phosphorylation of residue at Thr55 or Thr155 was reported to target p53 to degradation (32, 34, 44). On the contrary, phosphorylation of residue at Thr18 or Thr81 stabilizes p53 (29, 33, 45). In this study, we found that Cdc25B interacted with p53 and reduced the phosphorylation of several Ser/Thr residues on p53. Interaction between Cdc225B and p53 appeared to be important for dephosphorylating p53 as reduced interaction of the Cdc25B-CR mutant to p53 failed to dephosphorylate p53. Thus, our results suggest that binding of Cdc25B to p53 dephosphorylates p53 and prevents subsequent degradation by the proteasome. Interestingly, Cdc25B did not appear to target specific Ser/Thr residues of p53, as phosphorylations of at least four Ser/Thr residues were decreased by Cdc25B. Thus, Cdc25B might remove phosphate groups from p53 nonspecifically.

It was reported that DNA damage induced the phosphorylation of Ser15 and Thr18 residues on p53 (29). Phosphorylation of these two residues then disrupted its interaction with Mdm2 to prevent degradation by the ubiquitin-mediated pathway (46). Thus, dephosphorylation of these two residues by Cdc25B should promote p53 degradation. However, it was also reported that failed Thr55 phosphorylation of p53 was sufficient to induce nuclear localization of the protein and disrupt its interaction with Mdm2 in the absence of DNA damage where Ser15 and Thr18 were not phosphorylated (47, 48). Similarly, failed phosphorylation of Thr155 was reported to be sufficient for p53 stabilization (34). Thus, it is possible that dephosphorylation at Thr55 and/or Thr155 might dominate over Ser15/Thr18 to stabilize p53 by Cdc25B. Although the contribution of each Ser/Thr residue to p53 stability is unclear, it is likely that the combined effect of p53 dephosphorylation contributes to the overall stability of p53. Further experiments are required to clearly define the contribution of these residues on p53 stability.

## Experimental procedures

### Cell lines, culture condition, and growth analysis

The human normal lung fibroblast IMR90 was maintained in minimum essential medium (MEM; Gibco Thermo Fisher Scientific) containing 10% fetal bovine serum (FBS) and 1% penicillin/streptomycin. AD293 cells (human cervical epithelioid carcinoma cells), 293T cells (human embryonic kidney cells), A549 cells (human lung carcinoma cells), and human normal foreskin fibroblast (BJ) were maintained in Dulbecco's modified Eagle's Medium (Gibco Thermo Fisher Scientific) growth medium supplemented with 10% FBS and 1% penicillin/streptomycin. Both human colon cancer cell lines HCT116 WT and p53^−/−^ were maintained in McCoy’s 5A medium (Sigma). Human non-small-cell lung carcinoma H1299 cells were maintained in RPMI 1640 medium (Gibco). To determine cell proliferation, cells were digested with trypsin, stained with 0.2% trypan blue, and counted using a hemocytometer.

### Gene transfer into IMR90 cells

The CDC25B isoform 1 (P30305-2) was used in this study. Preparation of adenoviral delivery system for CDC25B, the AdEasyTM XL Adenoviral Vector System (Stratagene, http://www.stratagene.com/manuals/240009.pdf) was used following protocol described before (49). The Cdc25B catalytic inactive mutant (C487S and R493K, CR) was generated using KOD hot start DNA polymerase kit (EMD Millipore) using plasmid pBluescriptR-CDC25B as template. Primer pair 5’- ATCCTCATTTTCCACAGTGAATTCTCATCTGAGAAAGGG-3’ and 5’-CACATGCGGGGCCCTTTCTCAGATGAGAATTCACTGTG-3’ were used to generate CDC25B-CR mutation. The 6-His tagged wild-type and the CR mutant fragments were then PCR amplified from pBluescriptR carrying CDC25B-WT or –CR using primers (5’GGGGTACCATGCACCATCACCATCACCATGAGGTGCCCCAGCCGGA-3’ and 5’-CCGCTCGAGTCACTGGTCCTGCAG-3’), and subcloned into pShuttleCMV plasmid. The PacI-digested recombinant pShuttleCMV DNA was transfected into Ad293 cells using HyFectin Transfection Reagent Kit (http://www.hycell.com.tw/proa3-2.html). High titer viral stocks were prepared after repeated rounds of infection. Ad293 cells were used to determine viral titers. To infect IMR90, BJ1, H1299, and HCT116 cells, higher MOI values were required for CDC25B overexpression. To knock down p53 in IMR90 cells, the lentiviral delivery system was applied. All four lentiviral plasmids containing shRNA sequences against p53 were obtained from National RNAi core facility in Taiwan. The retroviral knockdown system was based on the RNAi Consortium (TRC) library pLKO.1 hairpin plasmid, the pCMV-ΔR8.91 packaging plasmid, and the pMD.G envelope plasmid (22) (Table S1). Lentiviral transductions were performed in 293T cells following the standard protocol.

### Immunoblotting analysis

Cells were washed with phosphate buffered saline (PBS, 137 mM NaCl, 2.7 mM KCl, 10 mM Na_2_HPO_4_, 1.8 mM KH_2_PO_4_, pH 7.4) and lysed in radioimmunoprecipitation assay (RIPA) buffer (20 mM Tris-HCl, 1% NP-40, 0.25% deoxycholic acid, 150 mM NaCl, 1 mM EDTA) containing 1 mM PMSF and 1x protease inhibitor (539134, Calbiochem). Total cell lysates were then collected by sonication of the cells followed by centrifugation at 16,000*g* for 5 min at 4 °C. Total cell lysates (30–50 μg) were separated by SDS-PAGE and then transferred onto nitrocellulose membranes. The membranes were hybridized with anti-p53 (Proteintech Group), anti-p53-S15 (Cell Signaling Technology), p53-T18 (GeneTex, Inc), p53-T55 (Abcam), p53-T81 (Cell Signaling Technology), p53-T155 (Santa Cruz Biotechnology, Inc), anti-Cdc25B (Cell Signaling Technology), anti-Cdk1 (Santa Cruz Biotechnology, Inc), anti-pCdk1-Y15 (Abcam), anti-p27 (GeneTex, Inc), anti-p21 (Cell Signaling Technology), anti-p16 (Proteintech Group), anti-pRB (S780, Cell Signaling Technology), or anti-RB (Proteintech Group). Horseradish-peroxidase-conjugated donkey anti-rabbit (GE Healthcare Life Sciences) or anti-mouse antibodies (Proteintech Group) were used as the secondary antibodies. The T-Pro LumiLong Plus Chemiluminescent Substrate Kit (T-Pro Biotechnology) was used to visualize the antibody-bound proteins.

### Cell cycle analysis

Cell cycle analysis was performed by monitoring the DNA content by propidium iodide staining. Cells were fixed in 70% cold ethanol followed by washing with PBS. The cells were then incubated with 1 mg/ml RNase A and 2 μg/ml propidium iodide at 4 °C for 30 min and then analyzed using a FACScan flow cytometer (BD Biosciences). About 10^6^ cells were acquired for analysis using Cell Quest software.

### Senescence-associated β galactosidase activity assay

Cells were washed with PBS and fixed in 4% formaldehyde and 0.2% glutaraldehyde for 5 to 10 min. The fixed cells were washed with PBS twice and then incubated with staining solution (40 mM citric acid, sodium phosphate, pH 6.0, 5 mM potassium ferrocyanide, 5 mM potassium ferricyanide, 150 mM NaCl and 2 mM MgCl_2_) containing 1 mg/ml 5-bromo-4-chloro-3-indolyl β-D-galactoside (X-Gal) at 37 °C for 12 to 16 h.

### Immunofluorescence assay

For the BrdU incorporation assay, cells were incubated with 40 μM BrdU for 16 h, washed with PBS, and fixed in 4% formaldehyde and 0.2% glutaraldehyde for 20 min. The fixed cells were washed with Tris buffered saline twice and then incubated with blocking solution (PBS contained 1% BSA (w/v), 0.1% TritonX-100) for 30 min. The cells were then incubated with 1.5 M HCl for 30 min and then washed with PBS. Visualization of BrdU incorporated cells was achieved by incubating the cells with antibody against BrdU (Cell Signaling Technology) followed by rhodamine-conjugated anti-mouse secondary antibody (Jackson ImmunoResearch Laboratories, Inc). For the DDR response assay, the cells were grown on slides and fixed with 4% formaldehyde at room temperature for 10 min. The fixed cells were then incubated in blocking solution for 1 h and in 1.5 HCl for 30 min. Anti-γ-H2AX antibody (GeneTex, Inc) was added and incubated at 4 °C for 16 h. The cells were then washed and incubated with Rhodamine-conjugated anti-mouse antibody (Jackson ImmunoResearch Laboratories, Inc) for 1 h. DAPI staining was conducted using mounting solution (Prolong Gold antifade reagent with DAPI), and the cells were visualized using fluorescence microscopy.

### Cdc25B phosphatase assay

Cdc25B phosphatase activity was measured using *p*-nitrophenyl phosphate (pNPP) as the substrate. The cells were lysed in RIPA buffer and total cell lysates were prepared. Cdc25B was immunoprecipitated by anti-Cdc25B antibody. The immunoprecipitates were washed twice with RIPA buffer resuspended in phosphatase assay buffer (20 mM Tris, pH 7.5, 5 mM MgCl_2_, 1 mM EGTA, 0.02% β-mercaptoethanol, 0.1 mg/ml bovine serum albumin). Phosphatase activities were then assayed with the addition of 1 mM DTT and 10 mM pNPP and incubated at 37 °C for 1 h. The reaction was stopped by NaOH, and the absorbance at 405 nm was measured using a spectrophotometer.

### Quantitative real-time polymerase chain reaction (qPCR)

The total RNA was extracted using GENEzol TriRNA pure kit (Geneaid Biotech, Ltd). First-strand cDNA was synthesized from 0.5 μg RNA with a RevertAid H Minus First Strand cDNA Synthesis Kit (Thermo Fisher Scientific). Real-time PCR was then conducted using SYBR Green Master Mix (Roche) kit in StepOne Real-Time PCR System (Applied Biosystems). The primer pairs for p53 were 5’-CCTGGATTGGCAGCCAGACT-3’ and 5’-GTTTCCTGACTCAGAGGGGG-3’. Primer pairs for GAPDH were 5’-GAAGGTGAAGGTCGGAGTCAA-3’ and 5’-CGTTCTCAGCCTTGACGGT-3’.

### Immunoprecipitation

IMR90 cells were lysed in immunoprecipitation (IP) buffer (50 mM Tris-base, 150 mM NaCl, 1 mM EDTA, 1% NP-40, pH 7.4) containing 1 mM PMSF and 1x protease inhibitor (539134, Calbiochem). About 300 μg cell lysates were incubated with 2 μg anti-p53 (Proteintech Group), anti-Mdm2 (Santa Cruz Biotechnology, Inc), or anti-IgG (Proteintech Group) antibody for 1 h, and then precipitated by adding Mag-Beads-Protein G (Tools, Taiwan) at 4 °C overnight. The beads were washed with PBS, and the precipitated proteins were eluted with 20 μl elution buffer (0.1 M Glycine, pH 2.0).

### p53 half-life determination

IMR90 cells were transfected with adenovirus carrying CDC25B at MOI = 30. The transfected cells were incubated with culture medium containing 20 μg/ml cycloheximide at 37 °C. Cells were harvested at various time points and cell lysates were prepared. Immunoblotting assays were then used to detect p53 levels.

### Statistical analysis

Student’s *t*-test was used to access whether the means of two groups are statistically different from each other. Values with *p* < 0.05, 0.01, or 0.001 were marked with ∗, ∗∗, or ∗∗∗, respectively.

## Data availability

All data are contained within the article.

## Supporting information

This article contains supporting information.

## Conflict of interest

The authors declare that there are no conflicts of interest.
