# Supplementary material for: CDC25B induces cellular senescence and correlates with tumor suppression in a p53-dependent manner
Source: J Biol Chem. 2021 Mar 18;296:100564. doi: 10.1016/j.jbc.2021.100564 (PMC8054198; doi:10.1016/j.jbc.2021.100564)
Supplement: Supplemental Figures S1–S4 and Table S1 [file mmc1.pdf]

CDC25B induces cellular senescence and correlates with tumor suppression in a p53-dependent manner

Ying-Chieh Chen[1], Hsi-Hsien Hsieh[1], Hsi-Chi Chang[1], Hsin-Chiao Wang[2], Wey-Jinq Lin[1,*], Jing-Jer Lin[1,2,*]

[1] Institute of Biopharmaceutical Sciences, National Yang-Ming University, Taipei, Taiwan

[2] Institute of Biochemistry and Molecular Biology, National Taiwan University College of Medicine, Taipei, Taiwan

[*] Corresponding Authors:

Jing-Jer Lin, Institute of Biochemistry and Molecular Biology, National Taiwan University College of Medicine, Taipei, Taiwan, 100. E-mail: jingjerlin@ntu.edu.tw;

Wey-Jinq Lin, Institute of Biopharmaceutical Sciences, National Yang-Ming University, Taipei, Taiwan, 112. E-mail: wjlin@ym.edu.tw

Running Title: CDC25B induces senescence

Key words: senescence, CDC25B, p53, tumor suppression

Table S1. shRNA sequences used to knockdown p53

Shp53	Clone ID	Target Sequence	Oligo Sequence	Region
#1	TRCN0000003753	CGGCGCACAG AGGAAGAGAA T	CCGGCGGCGC ACAGAGGAAG AGAATCTCGA GATTCTCTTC CTCTGTGCGC CGTTTTT	CDS
#2	TRCN0000003754	TCAGACCTAT GGAAACTACT T	CCGGTCAGAC CTATGGAAAC TACTTCTCGA GAAGTAGTTT CCATAGGTCT GATTTTT	CDS
#3	TRCN0000003756	CACCATCCAC TACAACTACA T	CCGGCACCAT CCACTACAAC TACATCTCGA GATGTAGTTG TAGTGGATGG TGTTTTT	CDS
#4	TRCN0000010814	GAGGGATGTT TGGGAGATGT A	CCGGGAGGGA TGTTTGGGAG ATGTACTCGA GTACATCTCC CAAACATCCC TCTTTTT	3’UTR

Supplementary figures

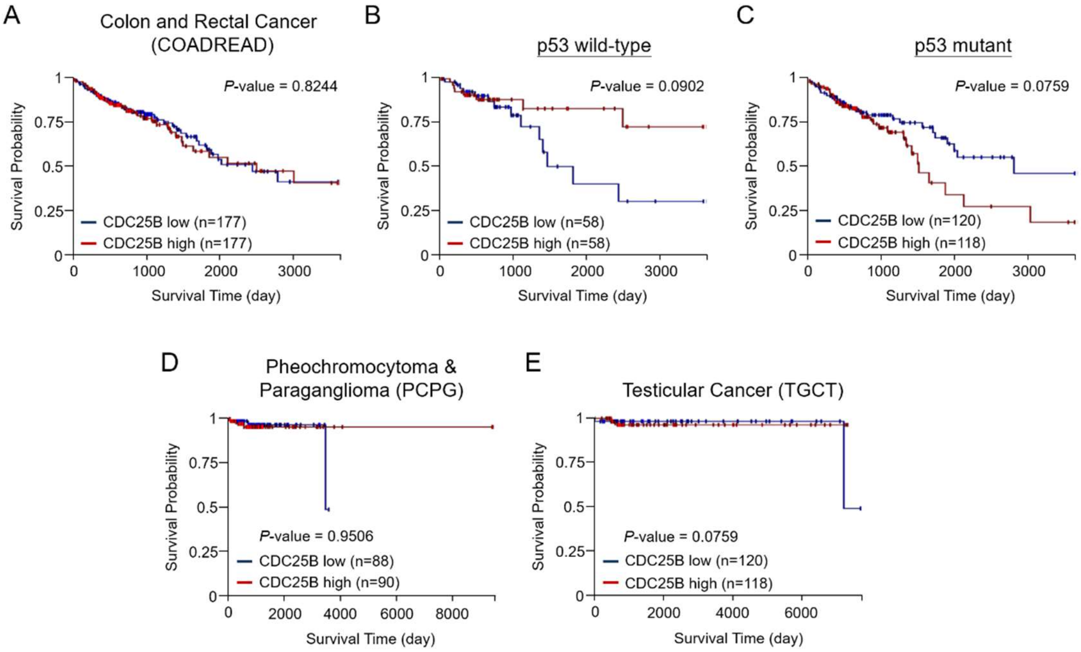

Figure S1. Kaplan-Meier survival curves of CDC25B expression levels and TP53 mutation status. The UCSC Xena (http://xena.ucsc.edu/) software was used to analyze dataset provided by the Cancer Genome Atlas program of National Cancer Institute (USA) (https://www.cancer.gov/about-nci/organization/ccg/research/structural-genomics/tcga). **(A)** Data from the TCGA Colon and Rectal Cancer (COADREAD) study were analyzed. In the analysis, CDC25B expression levels were broadly divided into two groups high (red) and low (blue). **(B)** The wild-type p53 cancers in the COADREAD study were analyzed. **(C)** Mutant p53 cancers in the COADREAD study were analyzed. **(D)** Data from the GDC TCGA Pheochromocytoma & Paraganglioma (PCPG) study were analyzed. In the analysis, CDC25B expression levels were broadly divided into two groups high (red) and low (blue). Since there is only one patient carrying p53 mutation, the results of wild-type p53 data were analyzed. **(E)** The GDC TCGA Testicular Cancer (TGCT) study was analyzed as above. Similarly, only the wild-type p53 data were analyzed due to the lack of p53 mutant patient. The statistic values (*P*) and number of patients (n) are indicated.

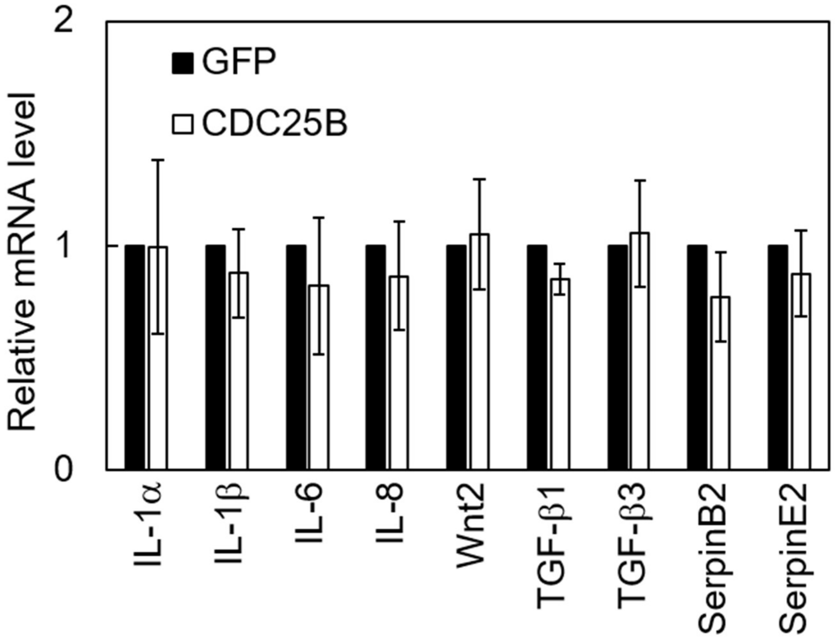

Figure S2. SASP in response to CDC25B overexpression. IMR90 cells were transduced with adenovirus carrying GFP or CDC25B at M.O.I.= 60. The cells were lysed and their mRNA analyzed by quantitative real-time PCR. The level of mRNA in GFP-treated cells was set as 1.

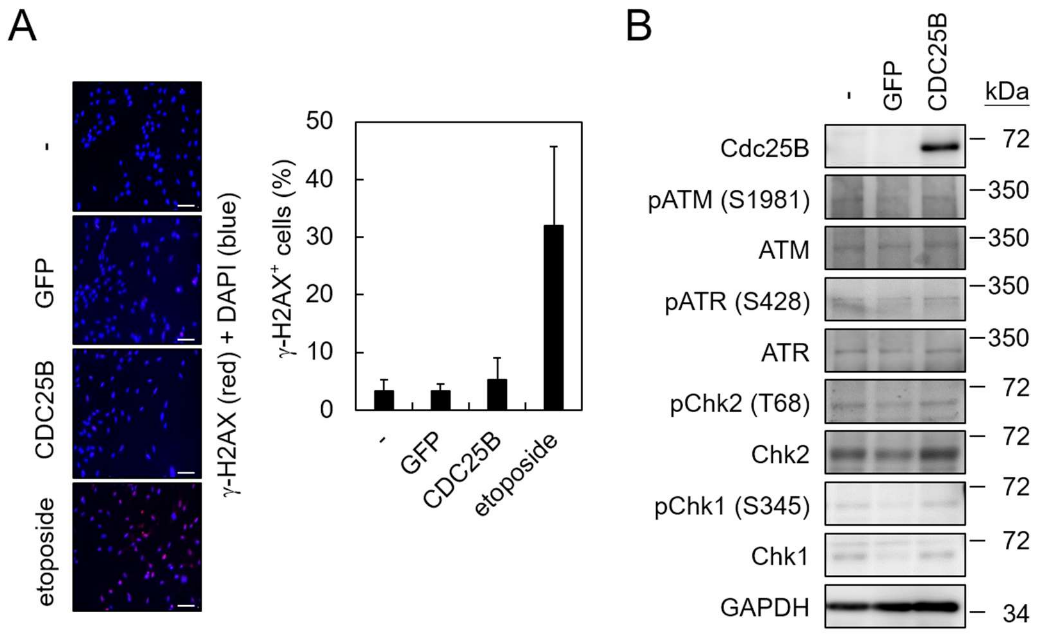

Figure S3. (A) IMR90 cells were infected with adenoviruses carrying CDC25B or GFP at M.O.I. = 60, the cells were fixed and stained with anti-γ-H2AX antibody (red) 3 days after infection. The nuclei were stained by DAPI (blue). As a positive control, cells were also treated with DNA damage agent etoposide. The percentage of the γ-H2AX-positive cells were presented. The scale bar represents 50 μm. **(B)** As above, cell extracts were prepared from the treated cells and analyzed for DDR activation by immunoblotting assays.

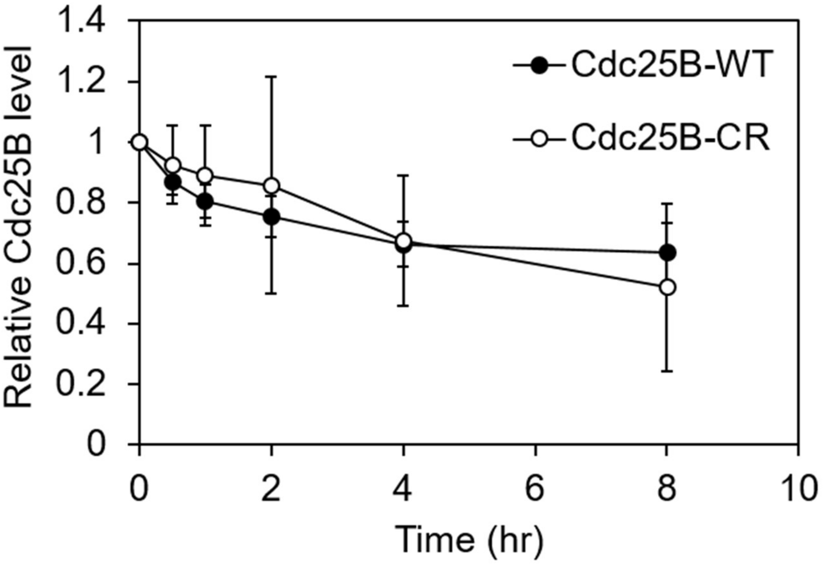

Figure S4. The CR mutation did not affect the apparent protein half-life of Cdc25B. As in Fig. 4B, quantification of the protein levels of Cdc25B-WT and Cdc25B-CR mutant were presented.
